# Periodontal disease and gastric and colorectal cancers: mechanisms and therapeutic perspectives

**DOI:** 10.3389/fcimb.2025.1699738

**Published:** 2025-12-15

**Authors:** Tianqi Wang, Hongliang Cao, Shengjie Ma, Zhen Wang, Heshi Liu, Haiyang Zhang, Quan Wang

**Affiliations:** 1 Department of Gastric and Colorectal Surgery, General Surgery Center, The First Hospital of Jilin University, Changchun, China; 2 Department of Urology II, The First Hospital of Jilin University, Changchun, China; 3 Department of Prosthodontics, Hospital of Stomatology, Jilin University, Changchun, China

**Keywords:** periodontal disease, gastric cancer, colorectal cancer, oral – gut microbiome axis, immune evasion, inflammation, therapeutic strategies

## Abstract

Periodontal disease (PD) is one of the most prevalent chronic oral diseases globally, characterized by chronic inflammatory responses in the gingiva and supporting periodontal tissues. Recent epidemiological and mechanistic studies have indicated that PD not only adversely affects oral health but is also significantly associated with gastric and colorectal cancers. This article reviews the potential link between PD and these gastrointestinal malignancies, exploring the underlying pathogenic mechanisms and potential strategies for preventing and treating gastric and colorectal cancers through the management of PD. Periodontal health management may represent an adjunct avenue for the prevention and control of gastrointestinal cancers; however, high-quality longitudinal and interventional studies are needed to clarify causality.

## 1 Introduction

Gastric and colorectal cancers, primarily referring to gastric cancer (GC) and colorectal cancer (CRC), represent a significant global health burden (Bray et al., 2024). These cancers originate from the mucosal epithelial cells of the stomach and colon, characterized by abnormal cell proliferation, local invasion, and distant metastasis. Despite differences in anatomical location, GC and CRC share commonalities in etiology, molecular pathogenesis, and the critical importance of early diagnosis for improving prognosis (Sung et al., 2021). GC and CRC are the fifth and fourth most common cancers worldwide, respectively, and are the third and second leading causes of cancer-related mortality (Dekker et al., 2019; Sundar et al., 2025). GC is particularly prevalent in East Asia and Eastern Europe, with associations to diet, Helicobacter pylori infection, and smoking, while CRC is more common in Western countries and Oceania, closely linked to unhealthy lifestyle choices (Karimi et al., 2014; Thrift and El-Serag, 2020; Li et al., 2021; Lin et al., 2021; Klimeck et al., 2023). The treatment of both cancers emphasizes a multidisciplinary approach, including surgical resection, systemic chemotherapy, molecular targeted therapies, and immunotherapy (Sexton et al., 2020; Biller and Schrag, 2021; Shin et al., 2023). Despite advancements in early diagnosis and comprehensive treatment, the overall prognosis for advanced GC and CRC remains poor, with low five-year survival rates, posing a severe threat to global public health.

Periodontal disease (PD) is one of the most prevalent chronic oral diseases worldwide, primarily characterized by chronic inflammation of the gingiva and supporting periodontal tissues, alveolar bone resorption, and tooth loss (Zhao et al., 2024). Its pathogenesis centers around periodontal pathogenic bacterial infection and abnormal host immune responses, influenced by multiple factors such as genetics, lifestyle, and overall health status (Li et al., 2025). With advancing research, PD is no longer regarded as merely a localized oral condition; growing evidence increasingly suggests its close association with a variety of systemic diseases (Li et al., 2022).

Recent years have seen a growing focus on the impact of PD on overall health. Numerous studies have shown that PD not only increases the risk of cardiovascular diseases, diabetes, respiratory conditions, and adverse pregnancy outcomes, but may also contribute to the development of gastrointestinal cancers, particularly GC and CRC, through chronic inflammatory responses and the systemic dissemination of pathogenic bacteria and their products (Sanz et al., 2018; Genco et al., 2020; Li et al., 2021; Baima et al., 2024a). Specifically, PD-associated pathogens such as *Fusobacterium nucleatum* (*F. nucleatum*) and *Porphyromonas gingivalis* (*P. gingivalis*) can spread via the bloodstream or digestive tract to colonize the gastrointestinal mucosa, inducing local immune imbalances, dysbiosis, and abnormal activation of carcinogenic signaling pathways, thereby promoting tumor initiation and progression (Nwizu et al., 2020). Therefore, it is essential to consider the impact of PD in the diagnosis and treatment of gastrointestinal tumors. This article reviews the epidemiological and pathogenic mechanisms linking PD to GC and CRC and discusses potential preventive and therapeutic strategies for PD.

## 2 Evidence linking periodontitis to GC and CRC

Recent epidemiological studies on the correlation between PD and gastrointestinal tumors have attracted widespread attention. Numerous systematic reviews, meta-analyses, prospective cohort studies, and case-control studies have been published, gradually forming a clearer research landscape. Overall, most studies support a significantly increased risk of GC and CRC in patients with PD; however, the strength and consistency of this correlation vary across different populations and research methodologies. The relevant studies are summarized in **Table 1**.

**Table 1:** Clinical studies have demonstrated the relationship between PD and GC and CRC.

Tumor	First author/year	Country	Sample size	Research type	Research objectives	Main outcomes
Gastric cancer	Chun-Han Lo (Lo et al., 2021), 2021	America	238	Prospective study	To assess the association between periodontal disease (PD) with the risk of gastric cancer (GC).	PD was associated with a 52% increased risk of GC(HR = 1.52; 95% CI = 1.13-2.04).
	Jinghua Sun (Sun et al., 2017), 2017	America, China	105	Prospective study	To assess the association between periodontal pathogen colonization and the risk of developing precancerous gastric lesions (PLGC).	Elevated periodontal pathogen burden predicted increased PLGC risk(P = 0.022).
	C Abnet (Abnet et al., 2001), 2001	America	28,868	Prospective study	To assess the association between tooth loss and GC risk.	Tooth loss was associated with a 80% increased risk of GC(RR = 1.80, 95% CI = 1.10–3.00).
	Christian C Abnet (Abnet et al., 2005), 2005	Finland	29,124	Prospective study	To assess the association between tooth loss and GC risk.	Tooth loss was associated with a 65% increased risk of GC(RR = 1.65, 95% CI = 1.05–2.49).
	Ngozi N Nwizu (Nwizu et al., 2017), 2017	America	65,869	Observational study	To assess the association between PD with the risk of new cancer cases in postmenopausal women.	PD is marginally associated with an increased risk of gastric cancer(HR = 1.58;95% CI = 0.94-2.67).
	Zengliang Ruan (Ruan et al., 2025), 2025	Sweden	5,888,034	Observational study	To assess the association between poor dental health and GC risk.	Poor dental health was associated with significantly higher risk of gastric cancer.
	Francisco José Nunes Aguiar (Aguiar et al., 2024), 2024	Brazil	9 studies	Meta-analysis	To assess the association between PD and GC risk.	PD increased the risk of developing GC by 17%(RR = 1.17;95% CI = 1.03-1.32).
	Q Wang (Wang et al., 2024), 2024	China	19 studies	Meta-analysis	To assess the association between PD and gastrointestinal cancer risk.	PD was associated with a 13% increased risk of GC(HR = 1.13, 95% CI = 1.01-1.26).
	X YIN (Yin et al., 2016), 2016	China	9 studies	Meta-analysis	To assess the association between tooth loss and GC risk.	Tooth loss was associated with a 44% increased risk of GC(RR = 1.44, 95% CI = 1.05–1.98).
Colorectal cancer	Dominique S Michaud (Michaud et al., 2018), 2018	America	7466	Prospective study	To assess the association of PD severity with CRC risk.	The risk of CRC is high among patients with severe periodontitis (HR = 2.12, 95% CI = 1.00 - 4.47).
	Manish Arora (Arora et al., 2010), 2010	Australia	15,333	Prospective study	To assess the association of tooth mobility with CRC risk.	Baseline periodontal diseasey was associated with increased risk of CRC (HR = 1.62, 95% CI:1.13-2.33).
	Jiyoung Ahn (Ahn et al., 2012), 2012	America	12,605	Prospective study	To evaluate the association between PD, serum antibody levels against *P. gingivalis*, and CRC–specific mortality.	Periodontitis-related mortality is elevated among individuals with CRC (RR = 3.58, 95% CI:1.15-11.16).
	Fatemeh Momen-Heravi (Momen-Heravi et al., 2017), 2017	America	77,443	Observational study	To assess the association between PD and colorectal cancer (CRC) risk in the Nurses’ Health Study (NHS) cohort.	Women with fewer teeth (<17 teeth) had a 20% increased risk of CRC (HR = 1.20, 95%CI = 1.04-1.39).
	Je-Ming Hu (Hu et al., 2018), 2018	China	212,974	Observational study	To assess the association between PD severity and CRC.	The cumulative risk of CRC in patients with periodontitis is significantly higher than in the control group (HR = 1.64, 95% CI = 1.50-1.80).
	Weiqi Li (Li et al., 2021), 2021	China	7 studies	Meta-analysis	To evaluate the association between PD and CRC risk.	PD significantly increased the risk of CRC by 44% (RR = 1.44;95% CI = 1.18-1.76).
	Kun Xuan (Xuan et al., 2021), 2021	China	14 studies	Meta-analysis	To explore the association between PD and CRC.	An association between PD and increased CRC incidence was found, PD patients were 21% (95%CI = 1.06, 1.38, I^2^ = 83.9%).

### 2.1 Epidemiological correlation between PD and GC

In terms of prospective cohort studies, Lo et al (Lo et al., 2021)found in a population follow-up that PD and tooth loss were significantly associated with an increased risk of GC. Similarly, Nwizu et al (Nwizu et al., 2017) in a subgroup analysis of the WHI Women’s Health Initiative cohort also observed a marginally elevated risk of GC associated with a history of PD. In a hospital-based case-control study conducted by Sun et al (Sun et al., 2017) at New York University, 105 participants were included, of whom 35 were diagnosed with precancerous lesions of gastric cancer (PLGC). The results showed that the levels of Treponema denticola (T. denticola) and Aggregatibacter actinomycetemcomitans (A. actinomycetemcomitans), two periodontal pathogens, were significantly higher in PLGC patients compared to controls, and the salivary microbiome diversity was notably reduced. Multivariable regression analysis indicated that higher levels of periodontal pathogen colonization and reduced microbiome diversity in dental plaque were independent predictive factors for an increased risk of precancerous gastric lesions. A prospective study conducted in China, which included 28,868 participants, found that tooth loss was associated with an increased risk of both gastric cardia cancer and non-cardia gastric cancer (Abnet et al., 2001). Another study conducted in Finland indicated a positive correlation between tooth loss and non-cardia gastric cancer, but no association with gastric cardia adenocarcinoma (Abnet et al., 2005). Ruan et al (Ruan et al., 2025) conducted a nationwide cohort study in Sweden, involving over 5.88 million adults, and innovatively introduced a sibling-control design. The study recorded 3,993 new cases of GC during an average follow-up of 6.4 years. Compared to individuals with good oral health, those with PD had an 11% increased risk of GC and a 25% increased risk of cardia cancer. Further dose-response analysis revealed that a lower number of remaining teeth was associated with a higher risk of GC, showing a clear negative correlation. The sibling-control analysis yielded consistent results with the main cohort study, further eliminating the effects of familial genetic and early environmental confounders, thereby strengthening the causal inference between PD and the increased risk of GC.

Several systematic reviews have also confirmed the aforementioned conclusions. Aguiar et al (Aguiar et al., 2024)conducted a meta-analysis, including nine high-quality original studies with a total of 3,754 patients. The results indicated that, compared to individuals with good oral health, patients with PD had a significantly higher incidence of GC. The study also analyzed the heterogeneity of different diagnostic methods and regions, finding that the association was more pronounced in Asian populations. Wang et al (Wang et al., 2024) performed a meta-analysis that included 19 cohort studies with approximately 16.62 million participants. The results showed a 13% increased risk of GC in PD patients compared to those with good oral health. The study also performed stratified analyses based on cancer location, PD type, and severity, revealing a more pronounced increase in the risk of GC and other gastrointestinal cancers in patients with severe PD. Furthermore, subgroup analysis showed that this correlation was robust across different countries and diagnostic methods, especially in populations with large sample sizes, long-term follow-up, and thorough adjustment for confounding factors. Sadighi et al (Sadighi Shamami et al., 2011) conducted a literature review and found that in five studies focusing on the upper gastrointestinal tract and GC, four reported that PD increased the risk of GC, showing a consistent epidemiological correlation. Some studies, even after controlling for confounders such as smoking and alcohol consumption, still indicated that PD was an independent risk factor for GC and upper gastrointestinal tumors. A study incorporated nine observational studies to assess the relationship between tooth loss and the risk of gastric cancer (Yin et al., 2016). The results suggested that tooth loss may be associated with an increased risk of gastric cancer. Subgroup analysis revealed that studies conducted in Asia showed a stronger association between tooth loss and gastric cancer, whereas studies in Europe and North America exhibited a weaker association. Furthermore, the study highlighted that traditional risk factors, such as smoking, alcohol consumption, and Helicobacter pylori infection, may act as confounding factors in this relationship.Fitzpatrick et al (Fitzpatrick and Katz, 2010) further pointed out in a literature review that the vast majority of epidemiological studies on GC found a significant positive correlation between PD and GC risk, although results varied somewhat across different studies.

### 2.2 Epidemiological correlation between PD and CRC

In prospective cohort studies, Momen-Heravi et al (Momen-Heravi et al., 2017) followed 77,443 women and found that patients with fewer than 17 teeth had a 20% increased risk of colon cancer compared to those with better tooth retention, with a more significant increase in the risk of rectal cancer. The study also showed that moderate to severe PD was associated with an elevated risk of CRC, further emphasizing the important link between periodontal health and CRC. Additionally, the study explored the combined effects of PD and tooth loss, finding that individuals with both periodontal bone loss and severe tooth loss had a significantly higher risk of developing rectal cancer. Michaud et al (Michaud et al., 2018) assessed the relationship between the severity of PD and the risk of various cancers, showing that patients with severe PD had a significantly higher overall cancer risk, with an increased trend for CRC, especially among edentulous individuals. In this group, the risk was more pronounced, with a more than two-fold increase in the risk of CRC in non-smokers. A prospective study based on the Swedish Twin Registry, which followed 15,333 participants for more than four decades, found a significant association between tooth mobility and an increased risk of CRC (Arora et al., 2010). Further analyses indicated that shared genetic factors and early-life environmental influences accounted for only part of this relationship and did not fully attenuate the observed risk. These findings suggest that PD may independently contribute to CRC, potentially through mechanisms involving heightened systemic inflammatory load, immune dysregulation, or the translocation of oral pathogens. Hu et al (Hu et al., 2018)conducted a retrospective cohort study that included 106,487 newly diagnosed PD patients and an equal number of controls, revealing that PD patients had a significantly higher cumulative risk of CRC compared to controls. Notably, the severity of PD was positively correlated with an increasing risk of CRC.

Several systematic reviews and meta-analyses have also provided high-level epidemiological evidence supporting this viewpoint. Li et al (Li et al., 2021) systematically reviewed seven relevant studies with a sample size of over 300,000 participants. The results showed that PD patients had a 44% increased risk of CRC, and this conclusion remained robust across multiple sensitivity analyses. Xuan et al (Xuan et al., 2021) conducted a meta-analysis that included 14 studies, finding that individuals with PD had a 21% increased risk of CRC compared to those with good oral health, although no significant association was found with mortality. Subgroup analysis further revealed that regional differences played a significant role in the correlation. The association was more pronounced in Europe and Asia, while no significant correlation was observed in North America.

Some studies have also focused on the association between specific periodontal pathogens and CRC. Baima et al (Baima et al., 2024a) conducted a retrospective analysis of multiple epidemiological datasets, systematically summarizing the latest data from large cohort studies in Europe, the United States, and Asia. The study highlighted that oral microbiome imbalances, including periodontal pathogens such as *F. nucleatum*, may play a promotive role in the development of CRC. The study also mentioned several large cohort studies with over ten years of follow-up, which found that individuals with progressively worsening periodontal health had a more pronounced increase in CRC risk, further reinforcing the epidemiological link between the two. A prospective cohort study based on the U.S. NHANES population, which followed 12,605 participants through 2006, demonstrated that moderate to severe periodontitis associated with *P. gingivalis* infection was significantly linked to an elevated risk of CRC mortality (Ahn et al., 2012). After adjustment for multiple lifestyle and demographic factors, individuals with PD exhibited an approximately threefold higher risk of CRC–related death. These findings suggest that chronic periodontal inflammation and exposure to periodontal pathogens may play an important role in the development and progression of CRC.

Research by Lauritano (Lauritano et al., 2017) and Pignatelli et al (Pignatelli et al., 2023) also emphasized the statistical association between PD, associated dysbiosis, and an increased risk of CRC.

## 3 Putative mechanisms by which periodontitis influences GC and CRC

Although existing observational studies suggest a correlation between PD and gastrointestinal tumors, the exact mechanisms underlying this association remain unclear. In this section, we primarily summarize the potential mechanisms through which PD may influence gastrointestinal tumors, which may include: migration and ectopic colonization of periodontal pathogens to the gastrointestinal tract, PD-induced local and systemic inflammatory states, and the impact of periodontal pathogens on immune evasion.

### 3.1 Migration and ectopic colonization of periodontal pathogens to the gastrointestinal tract

**Figure 1:**
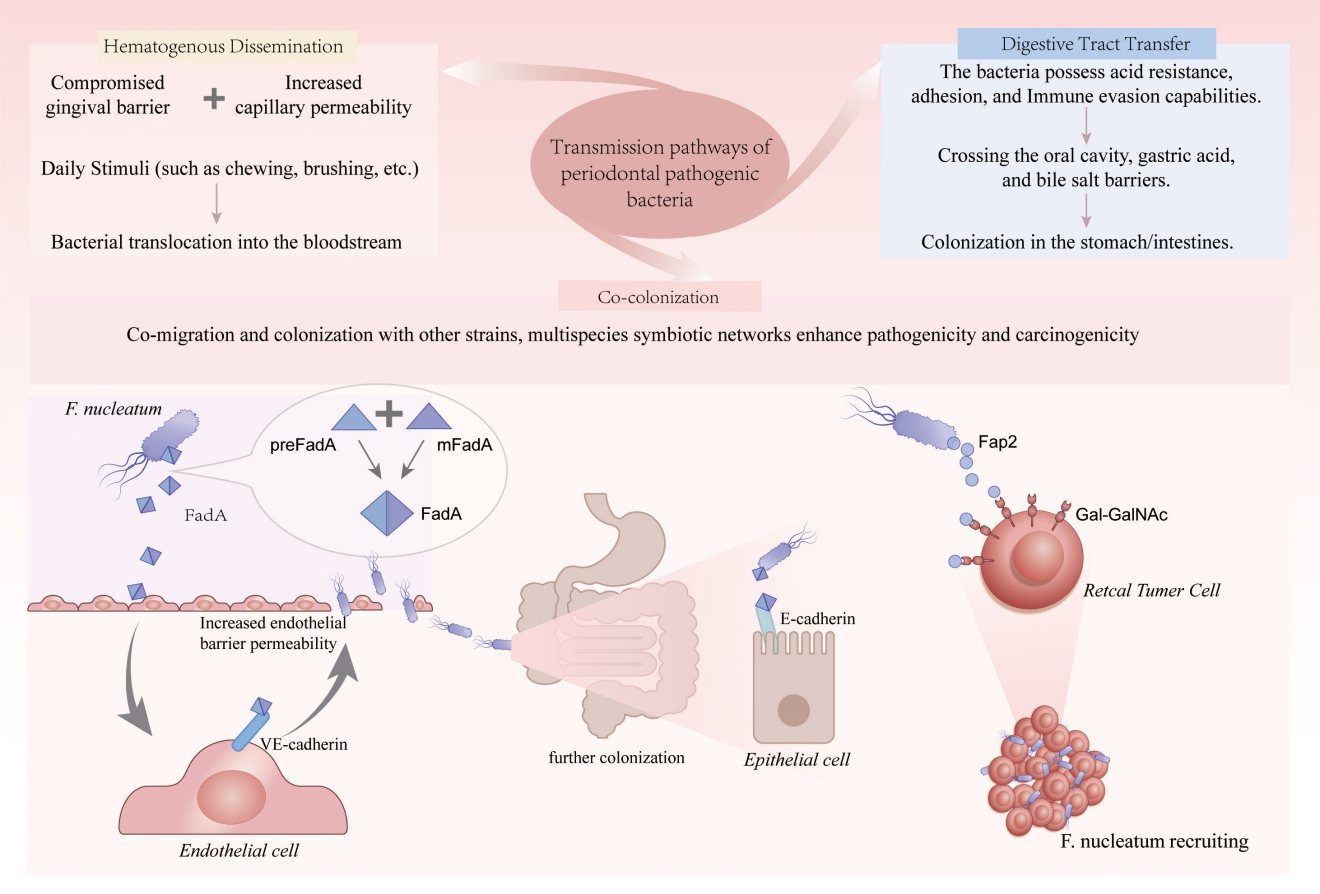
Transmission pathways and colonization mechanisms of periodontal pathogens in the gastrointestinal tract. Periodontal pathogens spread through three main routes: (i) hematogenous dissemination, where a compromised gingival barrier and increased capillary permeability allow bacterial translocation into the bloodstream during daily stimuli; (ii) digestive tract transfer, as bacteria with acid resistance, adhesion, and immune evasion capabilities cross oral, gastric, and bile salt barriers to colonize the stomach and intestines; and (iii) co-colonization, where co-migration with other microbes and multispecies symbiotic networks enhance pathogenicity and carcinogenicity. *F. nucleatum* promotes colonization via FadA-mediated binding to VE-cadherin and E-cadherin, increasing endothelial permeability and facilitating epithelial invasion, while its surface lectin Fap2 specifically recognizes Gal-GalNAc on CRC cells, enabling targeted enrichment in tumor tissues. *F. nucleatum*, *Fusobacterium nucleatum*; pre-FadA, precursor FadA; mFadA, mature FadA; VE-cadherin, vascular endothelial cadherin.

The oral cavity is the starting point of the human digestive tract and harbors up to 2,000 species of microorganisms, most of which are symbiotic, though some opportunistic pathogens have carcinogenic potential (Proctor et al., 2020). Under normal conditions, the oral microbiome maintains a dynamic balance, supporting both local and systemic health. The oral cavity and gastrointestinal tract are two anatomically and functionally connected regions of the digestive system, both rich in complex microbial communities. Recent studies have revealed that the oral microbiome and gastrointestinal microbiota interact through various physiological and pathological pathways (**Figure 1**).

Periodontal pathogens can migrate to the gastrointestinal tract through three main pathways: hematogenous dissemination, digestive tract transfer, and co-colonization (Sulaiman et al., 2024). PD is a chronic inflammatory oral disease that causes damage to the gingival epithelial barrier, increasing capillary permeability. In daily activities such as chewing, brushing, flossing, or periodontal surgery, bacteria in the oral cavity can enter the bloodstream through the damaged mucosa, leading to transient bacteremia (Emery et al., 2020). This bacteremia occurs occasionally in healthy individuals but is more frequent and intense in PD patients, with a stronger ability of the associated pathogens to colonize and cause disease. Gastrointestinal transfer is another significant route for periodontal pathogens. Recent studies have shown that periodontal pathogens are not limited to local infections; they possess strong acid tolerance, adhesion, and immune evasion abilities. These pathogens can successfully cross natural barriers such as the oral-pharyngeal region, gastric acid, and bile salts, colonizing the stomach or intestines, influencing the host’s immune response and microbiota, thereby inducing chronic inflammation, remodeling the tumor microenvironment, and promoting carcinogenesis (Sun et al., 2017). F*. nucleatum* exhibits strong acid resistance and stress response modulation, allowing it to survive in acidic environments, or under pathological conditions. Some studies have also indicated that these bacteria can enhance their resistance and colonization capabilities by being encapsulated in bacterial outer membrane vesicles (OMVs) (Surlin et al., 2020). In addition to hematogenous and digestive tract migration paths, periodontal pathogens can also migrate and colonize the gastrointestinal tract via the “co-colonization” mechanism, where they co-colonize with other microbial species, forming a multi-species symbiotic network that enhances their pathogenicity and carcinogenic potential. *F. nucleatum* plays a central role as a “bridging species” in this process. Its adhesion proteins, such as RadD and FadA, not only bind to host epithelial cells but also mediate bacterial-bacterial adhesion between early colonizers (such as streptococci) and later-stage pathogens (such as *P. gingivalis*), contributing to the formation of highly structured oral biofilms (Han, 2015). Studies have shown that co-culturing *F. nucleatum* with *P. gingivalis* significantly enhances their biofilm-forming ability and jointly stimulates intestinal epithelial cells to release inflammatory factors like IL-8, thereby inducing the formation of a tumor-associated inflammatory microenvironment (Nicolae et al., 2022). Additionally, multi-species co-migration exacerbates microbial imbalance through metabolic interactions, releasing toxic metabolites such as butyrate and hydrogen sulfide, which induce mucosal barrier disruption and immune activation, further driving the carcinogenesis process (Lam et al., 2023). Therefore, co-colonization not only enhances the survival and colonization ability of pathogens but also amplifies their pathological effects in the development of gastrointestinal tumors.

A key protein, FadA, is present on the surface of *F. nucleatum*. This protein exists in two forms: the precursor (pre-FadA) and mature (mFadA) versions, both of which can oligomerize to form an active complex (FadA). This complex is the molecular basis for *F. nucleatum*’s ability to adhere to and invade host cells (Xu et al., 2007). FadA specifically binds to vascular endothelial cadherin (VE-cadherin) on the surface of endothelial cells. VE-cadherin is a core molecule involved in intercellular junctions, helping maintain the integrity of the vascular barrier. Upon binding to VE-cadherin, FadA induces its internalization from the cell junctions, leading to a weakened endothelial barrier and increased permeability (Fardini et al., 2011). FadA not only allows direct invasion of host cells but also facilitates invasion through loosened intercellular junctions. This enables *F. nucleatum* to enter subendothelial tissue via the bloodstream, achieving ectopic colonization within the vasculature. Furthermore, the increased barrier permeability facilitates the “co-transfer” of other coexisting microorganisms, intensifying mixed infections, which may explain why *F. nucleatum* frequently appears in mixed infections in areas outside the oral cavity (Edwards et al., 2006). After traversing the endothelium, *F. nucleatum* binds to E-cadherin on the surface of local epithelial cells via FadA, promoting further colonization and activating host signaling pathways (such as β-catenin/Wnt), thereby inducing inflammatory responses, cell proliferation, and even tumorigenesis (Han, 2015).

Other studies have explored how *F. nucleatum* accurately targets and metastasizes to already-formed CRC. The surface protein Fap2 on *F. nucleatum* is a Gal-GalNAc-specific lectin that directly recognizes and binds to the high expression of the Gal-GalNAc sugar chain structure on CRC cells. Through tissue microarrays and cell experiments, it was found that the binding ability of *F. nucleatum* to CRC cells was closely related to the expression level of Gal-GalNAc, and this binding could be inhibited by O-glycanase treatment or competitive inhibition with free GalNAc. Furthermore, the Fap2-deficient strain showed a significant reduction in binding ability, indicating that Fap2 is a core molecule mediating this specific interaction. Immunofluorescence co-localization experiments also confirmed that Fap2-positive *F. nucleatum* colocalized highly with regions that expressed high levels of Gal-GalNAc. In conclusion, the interaction between Fap2 and Gal-GalNAc forms the molecular basis for the enrichment of *F. nucleatum* in CRC tissues (Abed et al., 2016).

### 3.2 The impact of PD-induced local and systemic inflammatory states on the development and progression of gastric and colorectal cancer

**Figure 2:**
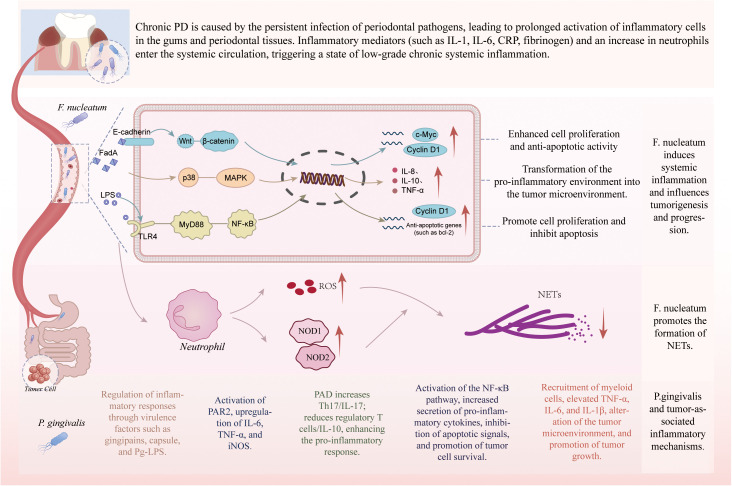
PD-driven inflammation in gastric and colorectal carcinogenesis. *F. nucleatum* induces systemic low-grade chronic inflammation and tumor progression through multiple pathways. By binding to E-cadherin via FadA, it activates Wnt/β-catenin, MAPK, and TLR4/MyD88/NF-κB signaling, leading to increased expression of oncogenes (c-Myc, Cyclin D1), pro-inflammatory cytokines (IL-8, IL-10, TNF-α), and anti-apoptotic genes, thereby enhancing proliferation, inhibiting apoptosis, and remodeling the tumor microenvironment. *F. nucleatum* also promotes neutrophil extracellular trap (NET) formation through ROS- and NOD1/2-mediated pathways. *P. gingivalis* contributes to tumor-associated inflammation via virulence factors (gingipains, capsule, Pg-LPS), activation of PAR2 and NF-κB signaling, induction of Th17/IL-17, suppression of Tregs/IL-10, and increased release of TNF-α, IL-6, and IL-1β, collectively fostering a pro-tumor inflammatory microenvironment. PD, Periodontal disease; *P. gingivalis, Porphyromonas gingivalis*; LPS, lipopolysaccharide; MyD88, Myeloid differentiation factor 88; TLR4, Toll-like receptor 4; PTK, protein tyrosine kinase; NF-κB, Nuclear factor kappa B; NETs, neutrophil extracellular traps; ROS, reactive oxygen species; Pg-LPS, porphyromonas gingivalis lipopolysaccharide; iNOS, inducible nitric oxide synthase; PAD, peptidylarginine deiminase; Tregs, regulatory T cells.

PD is an oral disease characterized by chronic inflammation. Due to the persistent infection by periodontal pathogens, the gums and periodontal tissues are continuously exposed to an inflammatory environment, leading to sustained activation of local inflammatory cells. The inflammatory response is not confined to the oral cavity but can induce a systemic low-grade chronic inflammatory state through various mechanisms (**Figure 2**). Studies have shown that, even after excluding confounding factors, patients with PD exhibit independent systemic inflammation, as evidenced by elevated plasma pro-inflammatory markers (such as IL-1, IL-6, CRP, and fibrinogen) and an increase in neutrophil count. These factors can enter systemic circulation, triggering systemic inflammatory responses and activating inflammatory signaling pathways in the bone marrow, immune system, and distant organs. Research indicates that *F. nucleatum* plays a key role in intestinal inflammation, primarily through its FadA adhesion protein. FadA binds to the EC5 domain of the epithelial cell adhesion molecule E-cadherin, and upon binding, it invades endothelial cells (Rubinstein et al., 2013). Furthermore, FadA activates the Wnt/β-catenin signaling pathway, which facilitates the translocation of β-catenin into the cell nucleus, initiating the expression of oncogenes (such as c-Myc and Cyclin D1) and upregulating the production of inflammatory cytokines and chemokines, thereby creating an inflammatory microenvironment conducive to tumor progression (Rubinstein et al., 2013; Zhang et al., 2019). Notably, protein tyrosine kinase (PTK) plays a crucial regulatory role in this signaling cascade, and the use of PTK inhibitors effectively blocks FadA-mediated E-cadherin phosphorylation, endocytosis, and subsequent nuclear translocation of β-catenin, along with its transcriptional activation (Le et al., 2002). In β-catenin knockout models, although FadA can still bind to E-cadherin and promote its endocytosis, no downstream gene activation is observed, further confirming the essential role of β-catenin in this signaling axis (Chan et al., 2002). Additionally, treatment with endocytosis inhibitors demonstrated that while the binding of FadA to E-cadherin and the phosphorylation of E-cadherin were unaffected, endocytosis was blocked, indicating that FadA-mediated tumor promotion and inflammation are dependent on both membrane binding and the endocytosis process (Bryant and Stow, 2004).

During the progression of colon cancer, the FadA protein of *F. nucleatum* activates two major signaling pathways. One of these pathways involves FadA’s adhesion to and invasion of human epithelial and endothelial cells, a process critical for the initiation of inflammation. The invasion by FadA leads to the upregulation of inflammation-related cytokines, particularly IL-8, IL-10, TNF-α, and Nuclear factor kappa B (NF-κB), which are regulated by the p38 MAPK signaling pathway (Mccoy et al., 2013; Rubinstein et al., 2019). Initially, these changes promote the formation of a pro-inflammatory microenvironment, which subsequently transitions into a tumor-promoting microenvironment, facilitating the progression of CRC. PD can also enhance the risk of gastrointestinal tumors by activating inflammation-associated pathways, leading to the upregulation of tumor-associated gene expression and enhancing cellular processes such as the cell cycle, anti-apoptotic mechanisms, invasion, and metastasis. *F. nucleatum* can activate the host’s Toll-like receptor 4 (TLR4)/Myeloid differentiation factor 88 (MyD88)/NF-κB signaling pathway through their lipopolysaccharide (LPS), promoting the release of pro-inflammatory factors. The activation of NF-κB leads to sustained inflammation, increased expression of cell cycle proteins and anti-apoptotic genes, thereby creating a conducive environment for tumorigenesis (Schwabe and Jobin, 2013; Gethings-Behncke et al., 2020; Mcilvanna et al., 2021; Fan et al., 2023).

In addition to inducing local and systemic inflammatory responses, periodontal pathogens can recruit inflammatory cells such as neutrophils. Kong et al (Kong et al., 2023) discovered that in CRC, neutrophil infiltration in tumor tissues is higher than in adjacent tissues, and the number of neutrophil extracellular traps (NETs) is significantly increased. Studies indicate that *F. nucleatum* can promote the massive formation of NETs by activating multiple signaling pathways in neutrophils, thereby driving the progression of CRC (Teles et al., 2020). Mechanistically, *F. nucleatum* first activates TLR4, promoting the generation of reactive oxygen species (ROS) within neutrophils. ROS, as downstream signaling molecules, drive the release of NETs. Meanwhile, *F. nucleatum* also upregulates the expression of intracellular receptors NOD1 and NOD2. The NOD1/2 signaling pathway, independent of ROS production, also plays a role in regulating NET formation. Blocking TLR4 or eliminating ROS significantly inhibits the release of NETs induced by *F. nucleatum*, and blocking NOD1/2 also notably reduces NET levels, suggesting that these two signaling axes are crucial regulatory pathways for *F. nucleatum*-mediated NET generation. The cooperation of both pathways significantly enhances the formation of tumor-associated NETs, providing a novel molecular basis for the initiation and progression of CRC.

P.gingivalis is a Gram-negative, anaerobic, rod-shaped oral bacterium and a key pathogen in chronic PD. The chronic inflammatory response triggered by its infection is one of the major pathways through which it contributes to tumorigenesis (De Jongh et al., 2023). Studies have shown that *P. gingivalis* can migrate from the oral cavity to various gastrointestinal sites, including the stomach and colon (Walker et al., 2018). Through its various virulence factors, such as gingipains, capsular structures, and *P. gingivalis* lipopolysaccharide (Pg-LPS), *P. gingivalis* modulates systemic inflammatory responses, disrupts microbial homeostasis, promotes tumor formation, and, to some extent, aids in evading the host’s innate immune defense mechanisms (Reyes, 2021). Research has indicated that gingipains, by activating the PAR2 receptor, significantly increase the expression of pro-inflammatory mediators, including IL-6, TNF-α, and inducible nitric oxide synthase (iNOS) (Liu et al., 2017). Zhao et al (Zhao et al., 2021) demonstrated that *P. gingivalis* can enhance the production of Th17 cells and IL-17 through its product, peptidylarginine deiminase (PAD), while reducing the production of regulatory T cells (Tregs) and IL-10, thereby amplifying the pro-inflammatory response. The NF-κB signaling pathway plays a critical role in tumor cell growth, survival, metastasis, and immune evasion. *P. gingivalis* activates the NF-κB pathway in host cells through its multiple virulence factors, leading to the secretion of cytokines such as TNF-αand IL-6. This not only promotes local inflammation but may also enhance tumor cell survival by inhibiting apoptotic signaling pathways (Lu et al., 2023). Studies have shown that *P. gingivalis* is significantly enriched in the fecal and tissue samples of CRC patients, and its high expression is associated with poor prognosis. In mouse models, *P. gingivalis* activates the NLRP3 inflammasome, promoting tumor initiation and progression. Furthermore, *P. gingivalis* recruits myeloid cells, alters the tumor microenvironment, and increases the production of pro-inflammatory cytokines, such as TNF-α, IL-6, and IL-1β, thereby further driving tumor growth (Wang et al., 2021).

### 3.3 The mechanisms by periodontal pathogens affect immune evasion

**Figure 3:**
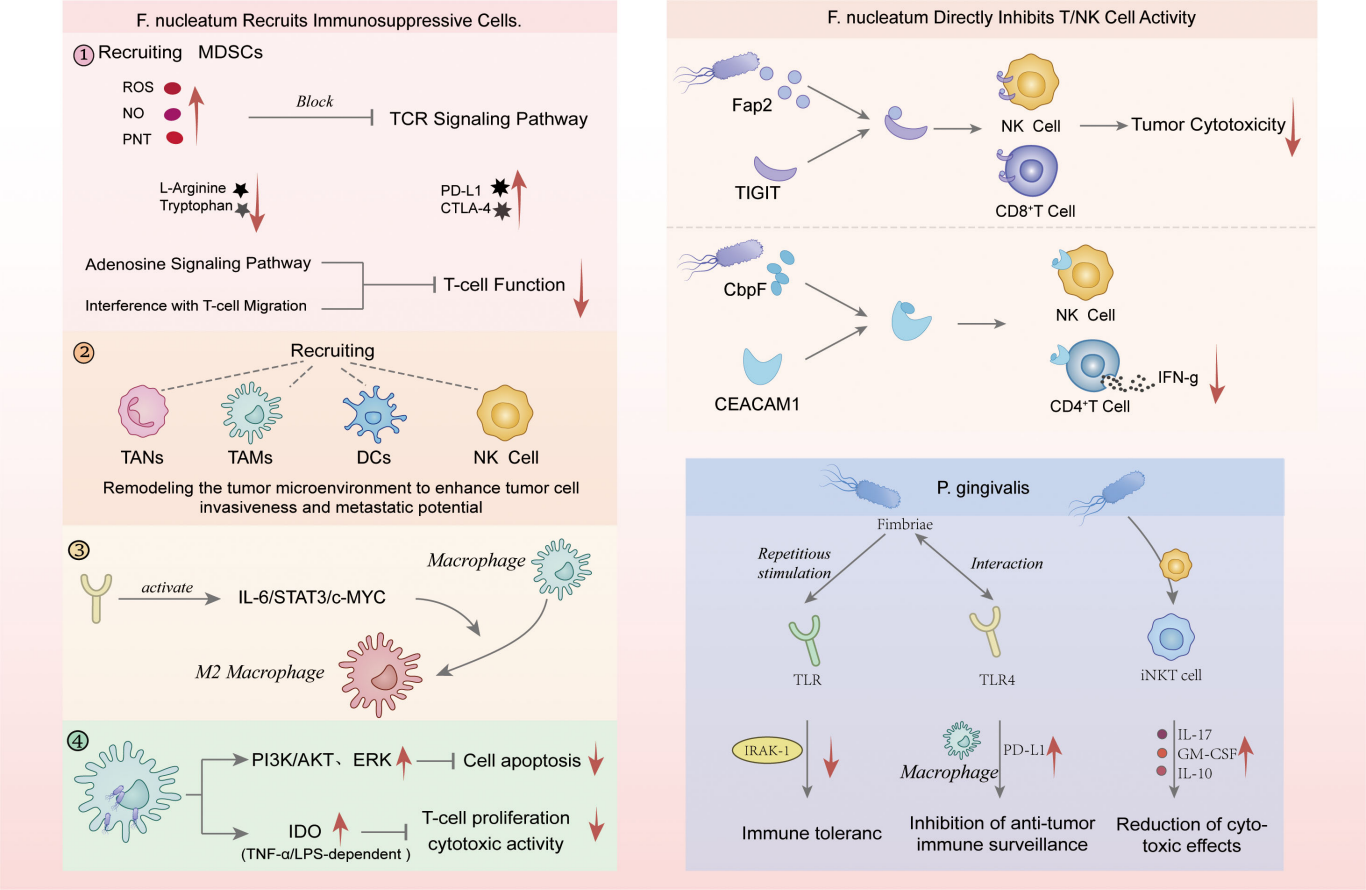
Mechanisms of immune evasion mediated by periodontal pathogens. *F. nucleatum* induces immune suppression by recruiting MDSCs that block TCR signaling, activate PD-L1/CTLA-4, and impair T-cell migration and function; by attracting TANs, TAMs, DCs, and NK cells to remodel the tumor microenvironment; by driving M2 macrophage polarization through IL-6/STAT3/c-MYC; and by activating PI3K/AKT and ERK pathways with IDO-dependent inhibition of T-cell proliferation and cytotoxicity. It also directly suppresses T/NK cell activity via Fap2–TIGIT and CbpF–CEACAM1 interactions, reducing cytotoxicity and IFN-γ secretion. *P. gingivalis* promotes immune evasion through fimbriae-mediated TLR modulation, IRAK-1 downregulation, PD-L1 upregulation in macrophages, and impaired iNKT cell activity, collectively enhancing immune tolerance and reducing anti-tumor responses. MDSCs, myeloid-derived suppressor cells; NO, nitric oxide; PNT, peroxynitrite; TCR, T-cell receptor; TAMs, tumor-associated macrophages; TANs, tumor-associated neutrophils; DCs, dendritic cells; IDO, indoleamine 2,3-dioxygenase; TILs, tumor-infiltrating lymphocytes; PAMPs, pathogen-associated molecular patterns; iNKT, invariant natural killer T.

Periodontal pathogens not only influence the inflammatory status of the body but also mediate tumorigenesis and progression by affecting the immune system, with *F. nucleatum* and *P. gingivalis* being the most prominent (**Figure 3**). Firstly, *F. nucleatum* exerts its effects by selectively recruiting or inhibiting various immune cells. It selectively recruits myeloid-derived suppressor cells (MDSCs) into the tumor microenvironment (Kostic et al., 2013a; Liang et al., 2022). MDSCs suppress immune responses through multiple mechanisms, including the production of ROS, nitric oxide (NO), and peroxynitrite (PNT), which disrupt the T-cell receptor (TCR) signaling pathway, the secretion of immunosuppressive cytokines (such as IL-10 and TGF-β), the expression of immune checkpoint molecules like PD-L1 and CTLA-4, and the further suppression of T-cell function by adenosine signaling and interference with T-cell migration (Yang et al., 2020). nucleatum also targets T-cell activity, and studies have shown that its abundance in CRC is negatively correlated with the density of CD3+ T and CD4+ T cells (Mima et al., 2015; Chen et al., 2018b). Shenker et al (Shenker and Datar, 1995) discovered that *F. nucleatum* can block the progression of human T cells at the G1 phase of the cell cycle through its secreted Fusobacterium inhibitory protein (FIP), thereby inhibiting T-cell proliferation without affecting activation. FIP also inhibits the expression of cyclins related to the late G1 phase, revealing the mechanism by which *F. nucleatum* induces immune suppression by interfering with the T-cell cycle.

Additionally, *F. nucleatum* can recruit tumor-associated macrophages (TAMs), tumor-associated neutrophils (TANs), regulatory dendritic cells (DCs), and NK cells, further remodeling the tumor microenvironment and enhancing the invasiveness and metastatic potential of tumor cells (Russo and Nastasi, 2022). Chen et al (Chen et al., 2018a) found that *F. nucleatum* activates the TLR4 signaling pathway to induce the polarization of TAMs to the M2 phenotype. This process relies on the IL-6/STAT3/c-MYC signaling cascade, with c-MYC primarily expressed in M2 macrophages, highlighting its crucial role in the formation of an immunosuppressive microenvironment. Butyrate, a short-chain fatty acid produced by *F. nucleatum* metabolism, significantly induces apoptosis in T cells and macrophages (Abe, 2012). Furthermore, Xue et al (Abe, 2012) revealed the survival mechanisms of *F. nucleatum* within macrophages and its connection to immune regulation. *F. nucleatum* can invade and proliferate to a limited extent within THP-1-derived macrophages, inhibiting host cell apoptosis through activation of the PI3K/AKT and ERK signaling pathways. Simultaneously, infection induces high expression of TNF-α and LPS-dependent indoleamine 2,3-dioxygenase (IDO), establishing an immunosuppressive microenvironment that impairs T-cell proliferation and cytotoxic activity. This mechanism allows *F. nucleatum*-infected macrophages to evade immune clearance, providing the conditions for persistent survival and spread within the host. In CRC, microbial dysbiosis and bacterial translocation can upregulate and maintain TGF-βlevels in pre-metastatic niches, reinforcing the immunosuppressive background (Wu et al., 2023). TGF-β further polarizes TANs to the pro-tumor N2 phenotype, whereas blocking TGF-βpromotes the accumulation of N1-TANs and upregulates chemotaxis/adherence and cytotoxic-related programs (such as CXCL1/2/5, ICAM-1), synergizing with CD8+ T cells to suppress tumor growth (Fridlender et al., 2009).

Additionally, *F. nucleatum* can bind to receptors on immune cells, thereby influencing immune responses. First, *F. nucleatum* can interact with the human inhibitory receptor TIGIT through its protein Fap2, thereby suppressing the tumor-killing activity of NK cells (Gur et al., 2015). This interaction prevents NK cells and tumor-infiltrating lymphocytes (TILs) from effectively attacking tumor cells. Banta et al (Banta et al., 2022) demonstrated that TIGIT and the activating receptor CD226 inhibit immune cell function by competing for the shared ligand CD155, thus suppressing the anti-tumor activity of CD8+ T cells. Furthermore, co-blocking TIGIT and PD-1 not only relieves the inhibition of CD226 by PD-1 but also restores CD226 functionality, significantly enhancing the anti-tumor response of immune cells. The study also discusses the role of TIGIT in regulating DCs; TIGIT promotes IL-10 production and inhibits IL-12, enhancing the immunosuppressive function of dendritic cells and inhibiting Th1 cell activation, thereby exacerbating immune suppression (Zhao et al., 2021). F*. nucleatum* can also activate another inhibitory receptor, CEACAM1, on NK and T-cell surfaces. The interaction between its surface protein CbpF and the N-terminal domain of CEACAM1 primarily activates CEACAM1, inhibiting the IFN-γ secretion response of CD4+ T cells and supporting immune evasion within the tumor microenvironment (Galaski et al., 2021). Quan et al (Jeon et al., 2023) found that CEACAM1 is selectively expressed in Tregs within tumors, and CEACAM1-positive Tregs accumulate as the tumor progresses. Notably, targeting CEACAM1 for selective depletion of Tregs can effectively remove highly suppressive Tregs from the tumor microenvironment, thereby improving the efficacy of immune therapies and providing a novel immune therapeutic strategy.

*P. gingivalis* can disrupt the host gut microbiome homeostasis by activating TLRs, leading to an imbalance that alters the direction of immune responses. Specifically, TLR activation induces the activation of innate immune signaling pathways, triggering the release of pro-inflammatory cytokines and chemokines. The signals mediated by TLRs may also weaken immune tolerance to beneficial bacteria and shift the immune response toward a direction exploited by pathogenic immune evasion strategies, thereby reducing effective clearing immune responses (Hajishengallis and Diaz, 2020). P*. gingivalis* further contributes to immune evasion through its fimbriae, which interact with host TLRs. Fimbriae are recognized by the immune system as pathogen-associated molecular patterns (PAMPs), activating TLR signaling pathways. However, repeated exposure to fimbriae leads to the downregulation of key signaling mediators such as IRAK-1, inducing immune tolerance and resulting in immune suppression (Hajishengallis et al., 2004). P*. gingivalis* also aids immune evasion by influencing macrophage function. *P. gingivalis* and its LPS activate the TLR-4 signaling pathway, promoting the upregulation of PD-L1 on the macrophage surface, thus inhibiting the immune surveillance and response of macrophages to tumor cells (Yu et al., 2019). Moreover, *P. gingivalis* modulates invariant natural killer T (iNKT) cell function, facilitating immune evasion and tumor progression in CRC. Studies have shown that *P. gingivalis* induces an anti-tumor iNKT cell phenotype by increasing the expression of IL-17, GM-CSF, and IL-10, which reduces iNKT cell-mediated tumor cell cytotoxicity. Additionally, *P. gingivalis* upregulates the protein CHI3L1, which interferes with iNKT cell lysis mechanisms and inhibits their activation through the PI3K/AKT signaling pathway, promoting immune evasion and tumor progression in CRC (Díaz-Basabe et al., 2024).

The gut microbiota not only influences the development of CRC by directly altering host immune responses and inducing chronic inflammation, but also plays a critical role in shaping the tumor microenvironment (El Tekle et al., 2024). Studies have shown that the gut microbiota can impact the functionality and composition of cancer-associated fibroblasts (CAFs) and the extracellular matrix (ECM), creating an immunosuppressive, tumor-promoting microenvironment. Specific gut microbes, particularly pathogenic species such as *F. nucleatum*, have been shown to affect CAF activity and function through multiple mechanisms. These microbes promote the secretion of pro-inflammatory cytokines (such as IL-6, TGF-β, and TNF-α) by CAFs, which enhances tumor cell proliferation and drives tumor growth and metastasis (Atarashi et al., 2011; Martin-Gallausiaux et al., 2018). Additionally, changes in the gut microbiota can directly influence ECM remodeling (Salvucci et al., 2022). Metabolic products from gut microbes, such as short-chain fatty acids (SCFAs), can modulate the role of CAFs in ECM remodeling, promoting the binding of cancer cells to the matrix, thereby enhancing tumor growth and metastatic potential (Singh et al., 2014). By modifying the composition of CAFs and ECM, the gut microbiota also plays a significant role in immune evasion.These findings suggest that the gut microbiota is not only involved in the immune evasion mechanisms of cancer but also contributes to the establishment of a tumor-promoting microenvironment by influencing CAFs and ECM in CRC.

## 4 Clinical approaches for impacting gastric and colorectal cancer progression through the treatment of PD

**Figure 4:**
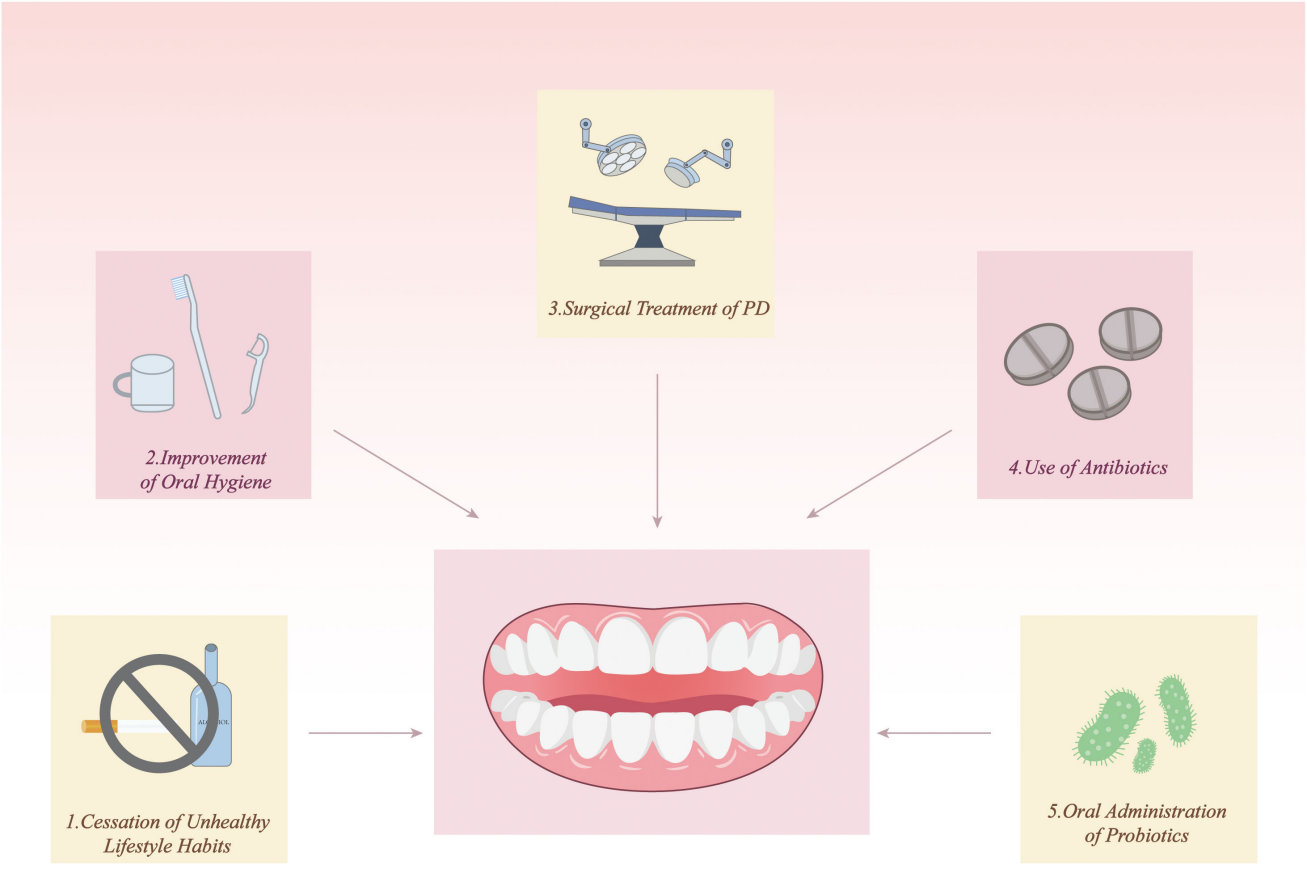
Clinical strategies for managing PD to reduce gastrointestinal cancer risk. Potential approaches include (1) cessation of unhealthy lifestyle habits such as smoking and alcohol consumption, (2) improvement of oral hygiene through regular brushing and interdental cleaning, (3) surgical treatment of periodontitis to remove infected tissues and restore periodontal structure, (4) use of antibiotics to control pathogenic bacteria, and (5) oral administration of probiotics to restore microbial homeostasis. These strategies aim to control periodontal inflammation and may contribute to lowering the risk of gastrointestinal tumor development.

PD can influence the development and progression of GC and CRC through multiple mechanisms. According to current research, the impact of PD treatment on gastrointestinal tumors can be achieved through various approaches, which are not limited to traditional dental treatments but also include emerging therapeutic strategies. These strategies include cessation of detrimental lifestyle habits, improved oral hygiene, surgical treatment of PD, the use of antibiotics, and oral probiotics (**Figure 4**).

### 4.1 Cessation of unhealthy lifestyle habits

PD is a chronic inflammatory disease caused by pathogenic bacteria in the oral cavity. Studies have shown that PD not only damages oral health but may also impact the development of gastrointestinal tumors, including GC and CRC, through systemic inflammation. By stopping unhealthy habits (such as smoking and alcohol consumption) and treating PD, the bacterial burden in the oral cavity can be reduced, thereby lowering systemic inflammation levels and indirectly influencing the occurrence of GC and CRC. Smoking is known to be one of the major risk factors for PD, as it promotes oral immune responses and inhibits the normal healing process of the gums, leading to periodontal tissue damage (Michaud et al., 2017). Harmful substances in tobacco not only exacerbate the inflammatory response in PD but also alter the composition of the oral microbiome, promoting the proliferation of harmful bacteria and accelerating the progression of PD. A study in Japan focused on the relationship between smoking and PD, involving 1,332 men, and found that smoking is an independent and significant risk factor for PD and tooth loss. Jennifer et al (Chang et al., 2021)examined the impact of smoking on clinical outcomes of non-surgical periodontal treatment. Smokers showed significantly less reduction in probing depth and less gain in clinical attachment level (CAL) after treatment compared to non-smokers, confirming the negative effect of smoking on non-surgical periodontal therapy. Building on this foundation, the research by Alqahtani et al (Alqahtani et al., 2023) further highlights the significant biochemical effects of smoking on PD. Their study found that smokers exhibited significantly higher calcium concentrations in their saliva compared to non-smokers, a change that may promote the formation of dental calculus and thus accelerate the progression of PD. While a negative correlation between salivary calcium and clinical indicators (such as CAL) was observed in non-smokers, no such correlation was found in smokers. This suggests that smoking may disrupt the typical relationship between calcium levels and PD by altering the oral environment (such as pH) and immune responses. These findings suggest that changes in calcium concentration could serve as a crucial marker for evaluating the progression of PD. Furthermore, studies have demonstrated that both serum and cortisol levels in smokers are significantly elevated compared to non-smokers, with cortisol levels showing a positive correlation with stress responses (Rahate et al., 2022). This indicates that smoking may exacerbate periodontal inflammation by increasing stress and cortisol secretion, thereby impairing the body’s anti-inflammatory responses. These results further support the multifaceted role of smoking in the onset and progression of PD, particularly through its negative impact on immune function and hormonal regulation. In summary, smoking not only alters the oral environment but also significantly influences the clinical manifestation of PD by increasing stress and hormonal disruptions. Smoking is also associated with the development and progression of GC and CRC. A pooled analysis of 10 cohort studies based in Japan found a significant association between smoking and an increased risk of CRC, with a dose-response relationship, and higher risks observed in men (Akter et al., 2021). Another study analyzed the relationship between smoking and survival in patients with GC and CRC, revealing that current and former smokers had worse prognoses compared to never-smokers. The study also found that smoking cessation improved survival outcomes, with the most significant improvement observed in patients who had quit smoking for more than 10 years (Walter et al., 2014).

Additionally, alcohol, like smoking, is also a significant factor contributing to PD. Chronic alcohol consumption can alter the composition of the oral microbiota, which in turn promotes the production of pro-inflammatory cytokines, leading to the degradation of the microstructure of periodontal bone (Pinto et al., 2020; Pinto et al., 2024). Studies have explored the relationship between alcohol abuse and PD, noting a significant correlation between alcohol consumption and the onset of PD, particularly among individuals from lower socioeconomic backgrounds who, despite consuming similar or lower amounts of alcohol, exhibit higher alcohol-related health risks (Oliveira et al., 2024). Cristine et al (Amaral Cda et al., 2008) found that alcohol dependence may weaken the host immune response, increasing susceptibility to infections, and it also affects bone metabolism, exacerbating periodontal tissue damage and leading to the development of PD. Other research has shown that while both smoking and alcohol consumption are associated with PD, alcohol may amplify the effects of smoking on periodontal health. The combined use of alcohol and smoking increases the risk of PD (Ryder et al., 2018). Gandhi et al (Gandhi et al., 2024) reported that long-term alcohol consumption not only alters the oral microbiota but also impairs immune function, thereby triggering systemic inflammation and increasing the risk of gastrointestinal tumors. Studies have found that moderate alcohol consumption (below 42g/day) does not significantly affect the risk of GC, while heavy drinking (>42g/day) is positively correlated with an increased risk of GC, indicating that heavy alcohol consumption significantly raises the risk of GC (Bouras et al., 2022). Research has also shown a significant dose-response relationship between alcohol intake and the risk of CRC and colorectal polyps (Fagunwa et al., 2017). Kim et al (Kim et al., 2019) analyzed 12 prospective cohort studies to explore the relationship between alcohol consumption and survival in CRC patients. The results revealed that light to moderate alcohol consumption before diagnosis was associated with a lower risk of all-cause mortality, while heavy drinking showed no significant survival benefits. For individuals with tobacco and alcohol dependence, some studies suggest dividing PD treatment into three parts: identifying the addiction stage, behavioral and pharmacological interventions, and population-level interventions. By using a combination of methods, these interventions help mitigate the impact of substance dependence on oral health (Kumar, 2020).

### 4.2 Improvement of oral hygiene

PD is a chronic inflammatory disease of the tissues surrounding the teeth caused by bacterial infection, primarily characterized by gingival inflammation and periodontal tissue destruction (Kinane et al., 2017). Conventional treatment focuses on controlling plaque and improving oral hygiene, with regular tooth brushing and the use of interdental cleaning tools serving as the foundation for preventing PD. In addition, methods such as providing personalized education for patients, setting practical and individualized goals for oral hygiene, and engaging in effective communication with patients can be combined with conventional treatments to control the progression of PD (Jönsson and Abrahamsson, 2020; Sälzer et al., 2020). Regular tooth brushing and flossing help reduce plaque accumulation, disrupt the formation of pathogenic biofilms, effectively lowering the incidence of gingivitis, and reducing the risk of periodontal tissue damage (Christou et al., 1998). However, the effectiveness of different interdental cleaning tools may vary. A study by Helen et al (Worthington et al., 2019), which reviewed 35 randomized controlled trials involving 3,929 participants, found that the use of dental floss or a toothbrush in conjunction with brushing may be more effective than brushing alone in reducing gingivitis and plaque, or both, with a toothbrush potentially being more effective than flossing. A novel artificial intelligence-based multimodal sensing toothbrush can optimize brushing behavior in real time, improving patient compliance with oral hygiene, and leading to better clinical outcomes (Li et al., 2024). Improving oral hygiene can effectively prevent and treat PD by reducing plaque accumulation, controlling the inflammatory response, and restoring the balance of the oral microbiome, thereby influencing the development and progression of gastrointestinal tumors.

### 4.3 Surgical treatment of PD

The primary goal of periodontal surgery is to remove the sources of infection in the affected periodontal areas and repair damaged tissues, thereby controlling the disease, promoting periodontal tissue regeneration, and ultimately restoring the function and stability of the tooth-supporting structures. Periodontal surgery can effectively remove calculus and plaque by excising the diseased gingiva and alveolar bone. This is because bacterial biofilms are the root cause of PD, and surgical treatment helps eliminate these pathogenic factors, thus slowing or even reversing the disease progression (Dommisch et al., 2020). In severe cases of PD, prolonged inflammation can lead to bone resorption, affecting the stability of the teeth. Techniques such as bone grafting may be required to repair the damaged alveolar bone structure (Graziani et al., 2017). Structurally, surgery can also remove diseased tissue adhering to the root surface through procedures such as curettage and root planing, restoring the connection between the teeth and the alveolar bone. This helps reduce the depth of periodontal pockets and improve tooth stability (Jepsen et al., 2020). Periodontal surgery can also reduce the risk of systemic infections by excising inflamed tissues, thus decreasing the opportunity for inflammatory mediators from PD to enter the bloodstream, which in turn lowers the risk of systemic diseases such as cardiovascular diseases, diabetes, and malignancies in periodontal patients (Neurath and Kesting, 2024). Postoperative antibiotic and antimicrobial therapy is often necessary to prevent infections and ensure effective treatment and accelerate healing. However, with the emergence of antibiotic resistance, there is an increasing need to develop new treatment approaches, such as antivirulence therapy and microbiome-based therapies.

### 4.4 Use of antibiotics

Oral pathogens are the primary cause of PD. Imbalance in the oral microbiota is one of the core features of PD. The proliferation of pathogenic bacteria disrupts the balance between the host and microbiota, leading to the formation of a pathogenic biofilm dominated by anaerobic bacteria (Larsson et al., 2022; Tonoyan et al., 2025). For patients with mild PD or recurrent cases, local antibiotic therapy can be applied (Jervøe-Storm et al., , 2022). Local antibiotic treatment directly targets the pathogenic bacteria within the periodontal pockets. Compared to systemic antibiotics, it achieves higher local concentrations at the treatment site, thereby more effectively controlling the local infection (Etienne, 2003). Since the drug is applied locally, systemic absorption is minimized, reducing the common side effects associated with systemic antibiotics and lowering the risk of bacterial resistance (Figuero et al., 2000). Local antibiotics can serve as an adjunct or alternative to surgical treatment, especially for patients who do not respond well to conventional periodontal therapy or for those with recurrent disease. By controlling the lesions locally, the dependency on surgery can be reduced (Lee et al., 2002; Patel et al., 2006). Several randomized controlled trials and systematic reviews have assessed the effectiveness of local antibiotic therapy in the treatment of PD. One study included multiple randomized clinical trials on smokers with chronic PD and evaluated whether local or systemic antimicrobial agents could improve clinical outcomes of non-surgical periodontal treatment. The results showed that local antimicrobial therapy improved the effectiveness of non-surgical periodontal treatment, while systemic antimicrobial agents did not significantly enhance the treatment outcomes (Chambrone et al., 2016). Paula et al (Matesanz-Pérez et al., 2013) summarized 56 studies on the role of local antibiotics in periodontal surgery, and the findings indicated that local application of antibiotics significantly improved patient outcomes. Moreover, in deep or recurrent periodontal sites, the use of local antibiotics was particularly beneficial in enhancing treatment effectiveness. Furthermore, studies have shown that local antibiotic application can provide additional benefits for smokers with PD (Rovai et al., 2016).

For patients with severe PD, systemic administration of antibiotics has been shown to produce more significant effects (Figuero et al., 2000; Keestra et al., 2015). Faggion et al (Faggion et al., 2014) evaluated whether systemic antibiotic therapy as an adjunct could effectively improve tooth survival in the treatment of PD. The study emphasized that although mechanical debridement remains the gold standard for PD management, the addition of systemic antibiotics may enhance therapeutic efficacy. Some studies have demonstrated that systemic antibiotics can improve clinical outcomes; however, evidence regarding their ability to increase long-term tooth survival remains insufficient. Other studies focusing on the clinical outcomes of non-surgical periodontal therapy combined with systemic antibiotics revealed that patients who received antibiotics during the early phase of treatment showed greater reductions in probing pocket depth and more pronounced improvements in clinical attachment level compared to those who received antibiotics after healing (Fritoli et al., 2015). Nonetheless, due to the high resistance of biofilms to antimicrobial agents and the lack of significant clinical improvements observed with antibiotics alone, systemic antibiotic therapy should be considered primarily as an adjunct following periodontal surgery for short-term suppression of oral pathogens, rather than for prolonged systemic use (Herrera et al., 2002). Yuan et al (Yuan et al., 2023)reported that long-term antibiotic use not only disrupts the balance of the gut microbiota but also impairs intestinal barrier integrity, increases intestinal permeability, and facilitates the translocation of pathogens and microbial products into the bloodstream, thereby affecting other tissues and organs, including periodontal tissues. Moreover, even after discontinuation of antibiotics, the gut microbiota did not fully recover, while the pathogenicity of the oral microbiome remained elevated. This suggests that the impact of antibiotics on microbial communities is long-lasting and may further worsen periodontal health over time (Ng et al., 2024).

### 4.5 Oral administration of probiotics

Unlike the traditional concept that disease arises from a single pathogen, modern periodontal research suggests that the transition from health to disease is primarily attributed to disruption of microbial community balance, namely microbial dysbiosis, which subsequently leads to dental plaque formation. PD is therefore regarded as a consequence of both microbial imbalance and microbially mediated disruption of host immune homeostasis (Zhang et al., 2022). This emerging paradigm has provided new therapeutic insights, emphasizing the restoration of microbial homeostasis associated with periodontal health. Recent studies have demonstrated that oral administration of probiotics may be applied in the treatment of PD (Myneni and Brocavich K, 2020). Probiotics exert their effects by modulating the oral microbiota, particularly through the inhibition of periodontopathogens, thereby reducing their growth and adhesion (Zidar et al., 2021). By altering the chemical and physical properties of the oral microbiome, probiotics exert beneficial effects on oral health (Teughels et al., 2007). Compared with pathogens, probiotics display stronger adhesion capacity, enabling them to effectively compete for binding sites, promote bacterial aggregation and co-aggregation, and facilitate the formation of new biofilms (Piwat et al., 2015; Takahashi, 2015). By competing for adhesion sites, nutrients, and growth factors, probiotics help restore microbial balance within the oral cavity, thereby protecting periodontal health. On one hand, probiotics mitigate dysbiosis by competing with periodontopathogens, reducing the overall immunogenicity of the microbiota.

In addition, probiotics can modulate immune and inflammatory pathways, alleviating the destructive inflammatory responses induced by PD and contributing to the maintenance of immune homeostasis (Homayouni Rad et al., 2023). Studies have shown that probiotic lactobacilli exhibit significant antimicrobial activity, primarily through the production of organic acids, hydrogen peroxide, and bacteriocins, which inhibit the growth of periodontopathogens (Sookkhee et al., 2001). During chronic PD, Lactobacillus species demonstrate antimicrobial activity against multiple periodontal pathogens, with the most pronounced inhibitory effect observed against *P. gingivalis*. Moreover, a direct correlation has been identified between reduced levels of lactobacilli and the severity of periodontal inflammation and tissue destruction (Kõll-Klais et al., 2005). Beyond decreasing the load of periodontal pathogens, probiotics can also effectively attenuate the host pro-inflammatory cytokine response (Szkaradkiewicz et al., 2014). Kobayashi et al (Kobayashi et al., 2017)reported that probiotics not only lower the levels of pro-inflammatory cytokines, such as IL-6 and TNF-α, in gingival tissues—thereby mitigating inflammation caused by oral pathogens—but also enhance local immune defense by upregulating defensin production. Thus, probiotics may be combined with antibiotics to both treat PD and reduce the risks of antibiotic resistance and systemic adverse effects (Rams et al., 2014). Despite the promising role of probiotics in periodontal therapy, current clinical studies demonstrate a degree of heterogeneity, likely attributable to factors such as strain selection, modes of administration, and individual variability (Petrariu et al., 2023). Future research should therefore aim to optimize probiotic formulations, explore combined applications with prebiotics or nanocarriers, and assess their long-term efficacy as well as their potential impact on systemic health.

## 6 Conclusions

This study first explored the relationship between PD and gastric and CRC. A substantial body of epidemiological evidence consistently indicates a significant association between PD and increased risk of GC and CRC. This relationship has been observed across diverse study designs, sample sizes, and populations, and in some studies, a dose–response pattern has been identified, suggesting a potential causal link. Current evidence indicates that PD may contribute to tumor initiation and progression through various mechanisms. These mechanisms include the establishment of the oral–gastrointestinal axis, migration and ectopic colonization of periodontal pathogens, sustained chronic inflammation, and immune modulation and evasion. Specifically, periodontal pathogens such as *F. nucleatum* and *P. gingivalis* can disseminate to the gastrointestinal tract via the bloodstream or digestive system, where they localize and induce immune dysregulation, thereby activating carcinogenic signaling pathways (Alon-Maimon et al., 2022). Research has shown that these pathogens are not only capable of crossing the oral–gastrointestinal barrier, but also contribute to tumorigenesis by promoting gut dysbiosis, stimulating local immune responses, and driving inflammatory processes, thus creating a tumor-promoting microenvironment (Wang and Fang, 2023). Moreover, persistent chronic inflammation represents another key mechanism linking PD to gastrointestinal cancers. The continuous accumulation of inflammatory mediators and the sustained activation of immune cells foster an environment conducive to tumor development. Immune evasion mechanisms also play a central role in the pathogenesis of gastrointestinal tumors in the context of PD. Periodontal pathogens can disrupt the host’s immune response via various pathways, facilitating immune escape within the tumor microenvironment (Kostic et al., 2013a). For instance, *F. nucleatum* adheres to host cells through its FadA protein, which activates carcinogenic signaling pathways, such as Wnt/β-catenin, further exacerbating local immune suppression and impairing T-cell function. This immunosuppressive environment creates conditions that promote tumor progression. Through these immune-modulating mechanisms, periodontal pathogens not only facilitate tumor initiation but may also impact metastasis and disease prognosis (Mysak et al., 2014).

The modifiable and treatable nature of PD presents a viable avenue for reducing cancer risk through multiple interventions. Strategies such as enhancing oral hygiene, controlling smoking and alcohol consumption, administering systematic periodontal treatment, and incorporating probiotics have all demonstrated efficacy in reducing the chronic inflammation associated with PD, thereby mitigating the risk of gastrointestinal cancers. Evidence indicates that smoking and alcohol consumption not only intensify the inflammatory response in PD but also disrupt the oral microbiota, fostering the growth of pathogenic bacteria that accelerate periodontal disease progression (Apatzidou, 2022). Notably, smokers exhibit elevated calcium concentrations in their saliva, a shift that may facilitate dental calculus formation and exacerbate the progression of PD. By improving the oral environment through smoking cessation and alcohol moderation, the risk of gastrointestinal cancers may be indirectly diminished. The use of probiotics presents another promising intervention. Probiotics help modulate the oral microbiota, suppressing the growth of periodontal pathogens and mitigating their detrimental effects on the host immune system, thereby promoting oral health. Studies have shown that oral probiotics can restore microbial balance, reduce inflammation driven by PD, and lower cancer risk linked to chronic inflammation (Nie et al., 2023). As research into probiotics’role in oral health continues, they may emerge as a novel therapeutic target for preventing gastrointestinal cancers.

A more compelling aspect is the enrichment of specific periodontal pathogens in gastrointestinal tumor tissues and their involvement in immune evasion. This suggests that modulating the oral microbiota could be a promising approach for the targeted prevention and management of gastrointestinal cancers. Pathogens adhere to host cells via surface proteins, triggering carcinogenic signaling pathways that promote tumor initiation and progression (Akbari et al., 2024; Zhou et al., 2024). These findings support the potential of oral microbiota regulation as a novel and targeted strategy for preventing gastrointestinal cancers. Future research should prioritize investigating how oral health management, particularly through microbial modulation, can influence tumor initiation and progression.

Despite robust evidence linking PD with an elevated risk of GC and CRC, several limitations within the current body of research merit attention. Firstly, much of the available evidence stems from observational studies, which, although valuable, are inherently constrained by the potential influence of confounding factors such as lifestyle choices, socioeconomic status, and the presence of other comorbid conditions. These variables may affect the observed associations and complicate the establishment of causal relationships. Secondly, there is substantial heterogeneity in the diagnostic criteria, classification systems, and methodologies employed to assess PD across studies. This lack of consistency undermines the comparability of findings and limits the generalizability of conclusions. Furthermore, variations in oral hygiene habits, dietary patterns, and microbiome composition among different populations may contribute to geographic differences in study outcomes. Finally, while the proposed mechanisms linking PD to tumor initiation and progression—such as microbial translocation, chronic inflammation, and immune modulation—are biologically plausible, they remain largely theoretical. Further investigation is needed to clarify these mechanisms in greater detail and to address the methodological challenges inherent in the existing studies.

Large-scale, multicenter, prospective cohort studies are essential to further establish the causal relationship between PD and GC or CRC. These studies should integrate long-term follow-up data and apply molecular biology techniques to explore the specific mechanisms by which periodontal disease contributes to the development of gastrointestinal cancers. High-quality randomized controlled trials will be crucial to assess the impact of periodontal interventions on cancer risk and prognosis. In particular, the efficacy of periodontal interventions in early cancer detection and personalized treatment strategies must be rigorously evaluated through clinical trials to provide evidence-based support for cancer prevention and control.

Moreover, fostering interdisciplinary collaboration among disciplines such as dentistry, oncology, immunology, and microbiology will be pivotal in integrating oral health management into comprehensive strategies for the prevention and control of gastrointestinal cancers. This collaborative effort will facilitate early interventions, preventive treatments, and personalized medical approaches, ultimately providing more effective strategies to reduce the incidence of gastrointestinal cancers.
